# Supplementary material for: Joint reconstruction of multi-contrast relaxation-diffusion imaging
Source: bioRxiv. 2026 Oct 26:2026.10.21.760608. Preprint. [Version 1] doi: 10.64898/2026.10.21.760608 (PMC13733696; doi:10.64898/2026.10.21.760608)
Supplement: Supplementary file 1 [file media-1.pdf]

Supplementary Material

Supplementary Figures S1 and S2 provide the FA and MD maps for the retrospective HCP multi-shell acceleration experiment. Supplementary Figures S3–S5 provide FA, MD, and DTI orientation maps at coronal slice $y = 53$ across the complete 3×3 b-value/TE grid in the multi-shell multi-TE experiment.

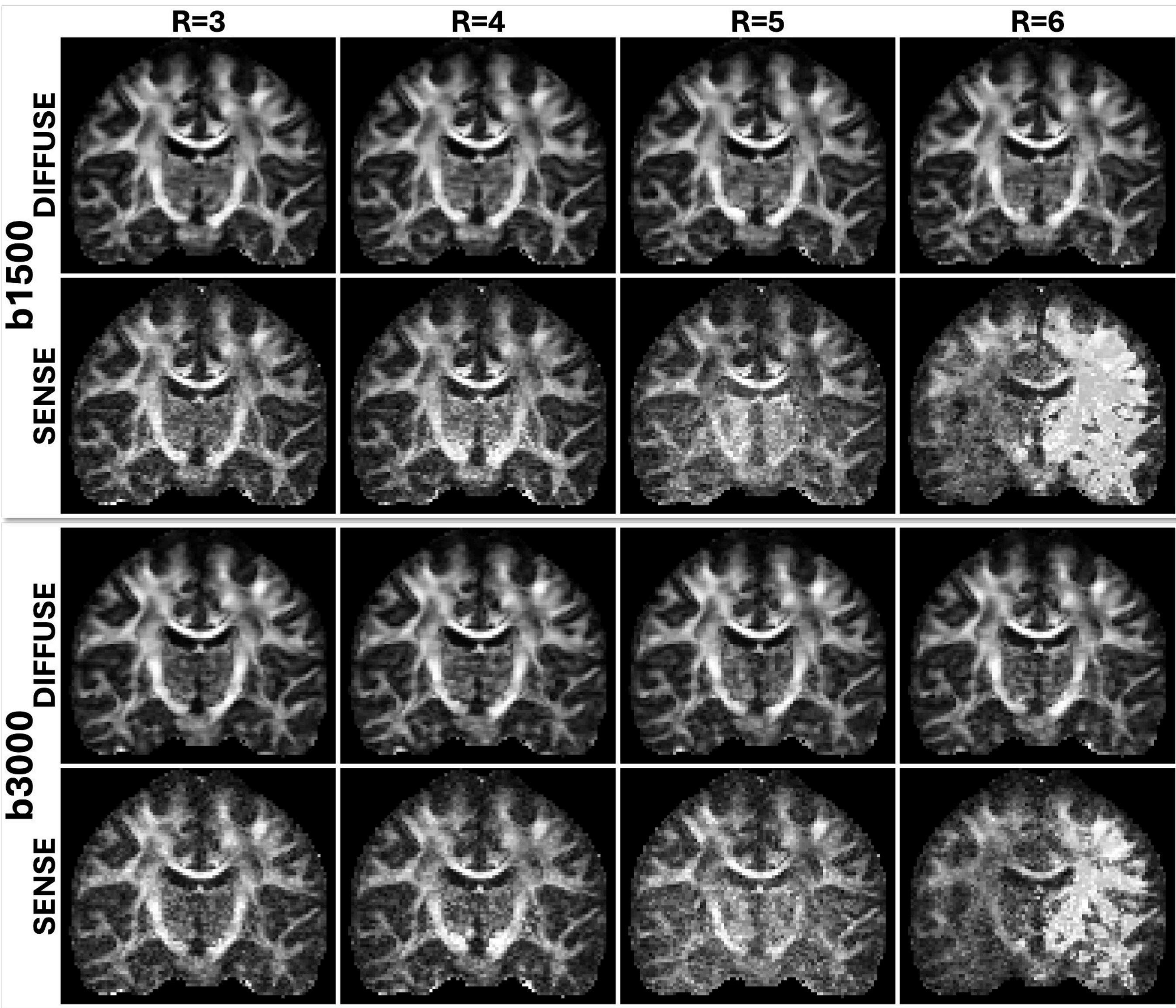

Fig. S1. Fractional anisotropy (FA, scale 0–1) for retrospective HCP multi-shell acceleration. Maps are arranged by acceleration factor and shell, with SENSE and DIFFUSE compared under matched tensor-estimation settings. DIFFUSE reduces visible aliasing-driven contamination of FA at $R = 5$ and $R = 6$.

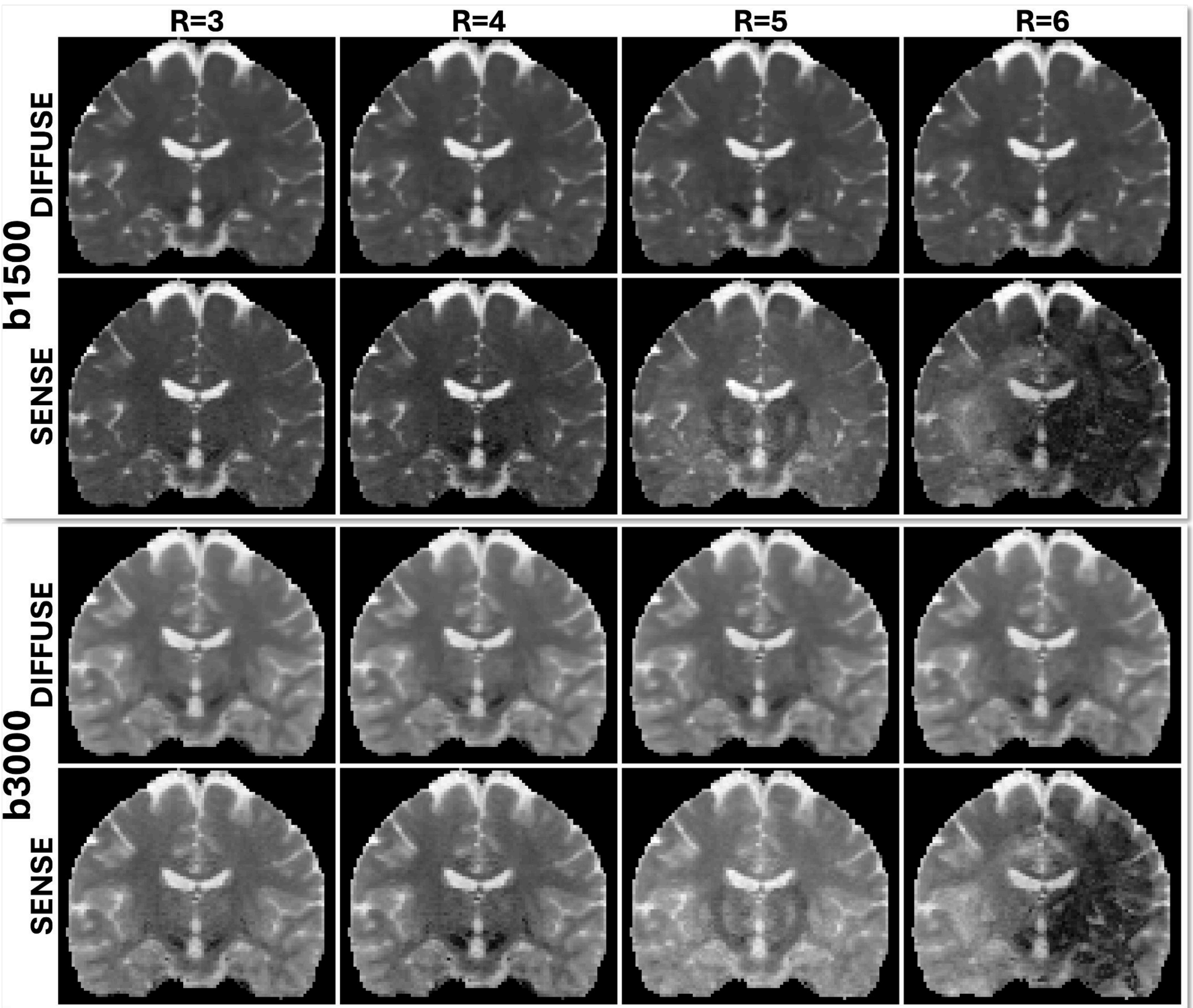

Fig. S2. Mean diffusivity (MD, scale 0–0.003 **mm^2/s) for retrospective HCP multi-shell acceleration. Maps are arranged by acceleration factor and shell, following the same layout as the DWI panels and DTI orientation maps.**

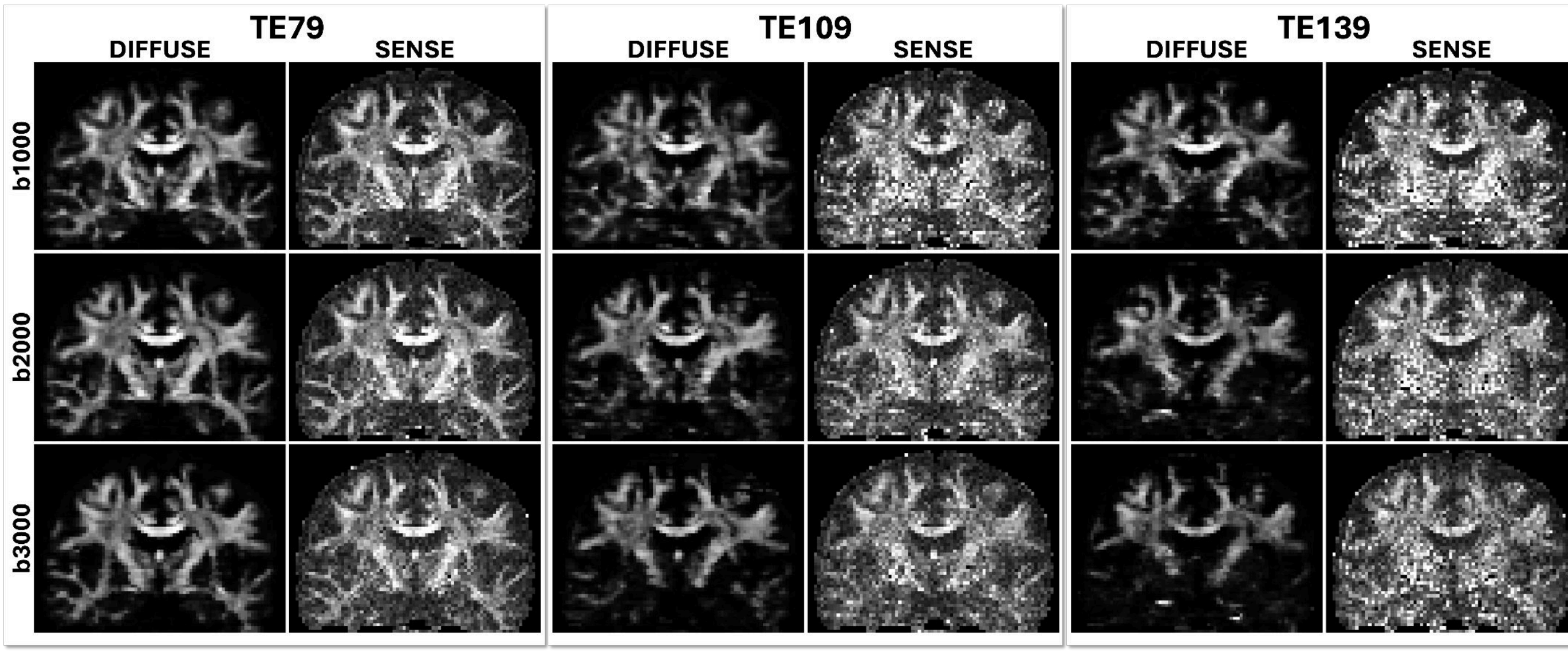

Fig. S3. Fractional anisotropy (FA, scale 0–0.8) maps at coronal slice $y = 53$ across the complete 3×3 b-value/TE grid in the multi-shell multi-TE experiment. Rows show $b = 1000$, 2000, and 3000 s/mm^2. Column pairs show TE values of 79, 109, and 139 ms, with DIFFUSE and SENSE displayed side by side within each pair.

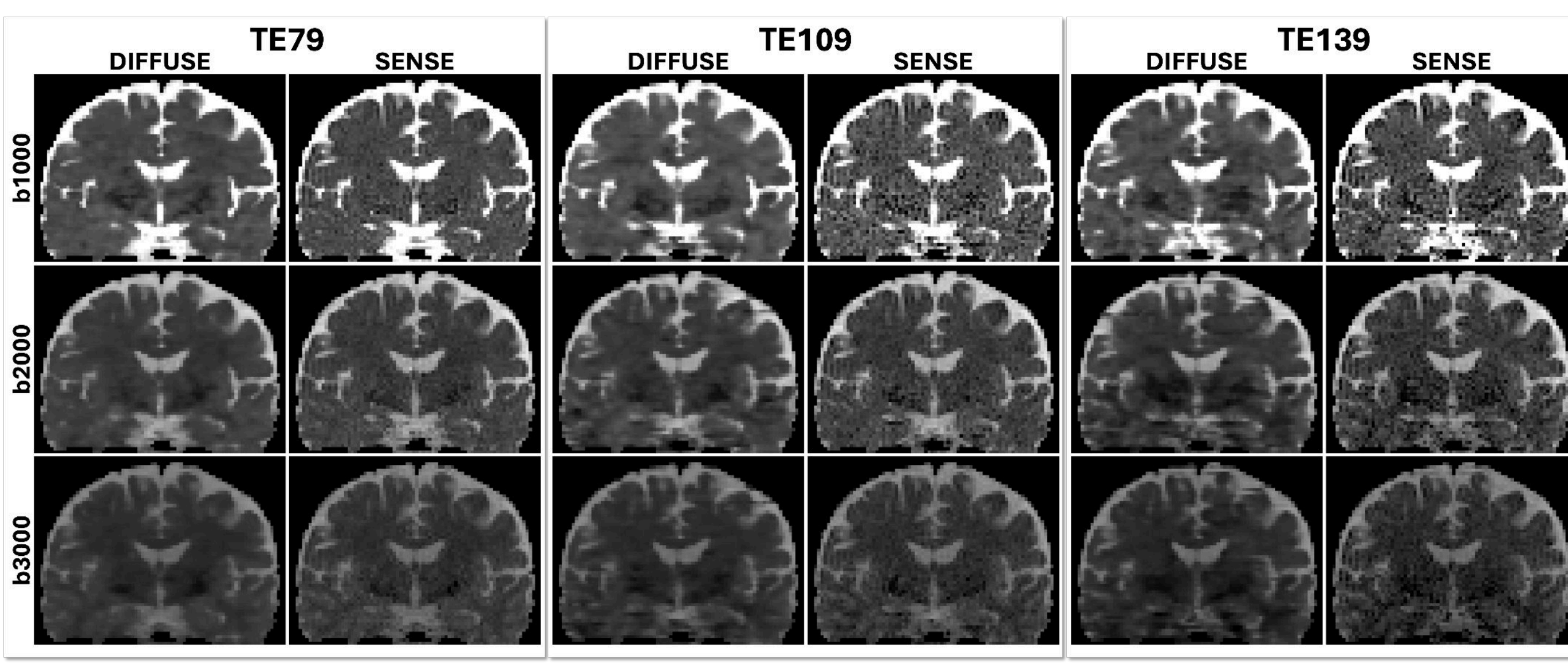

Fig. S4. Mean diffusivity (MD, scale 0–2.5×10^{-3} mm^2/s) maps at coronal slice $y = 53$ across the complete 3×3 b-value/TE grid in the multi-shell multi-TE experiment. Rows show $b = 1000$, 2000, and 3000 s/mm^2. Column pairs show TE values of 79, 109, and 139 ms, with DIFFUSE and SENSE displayed side by side within each pair.

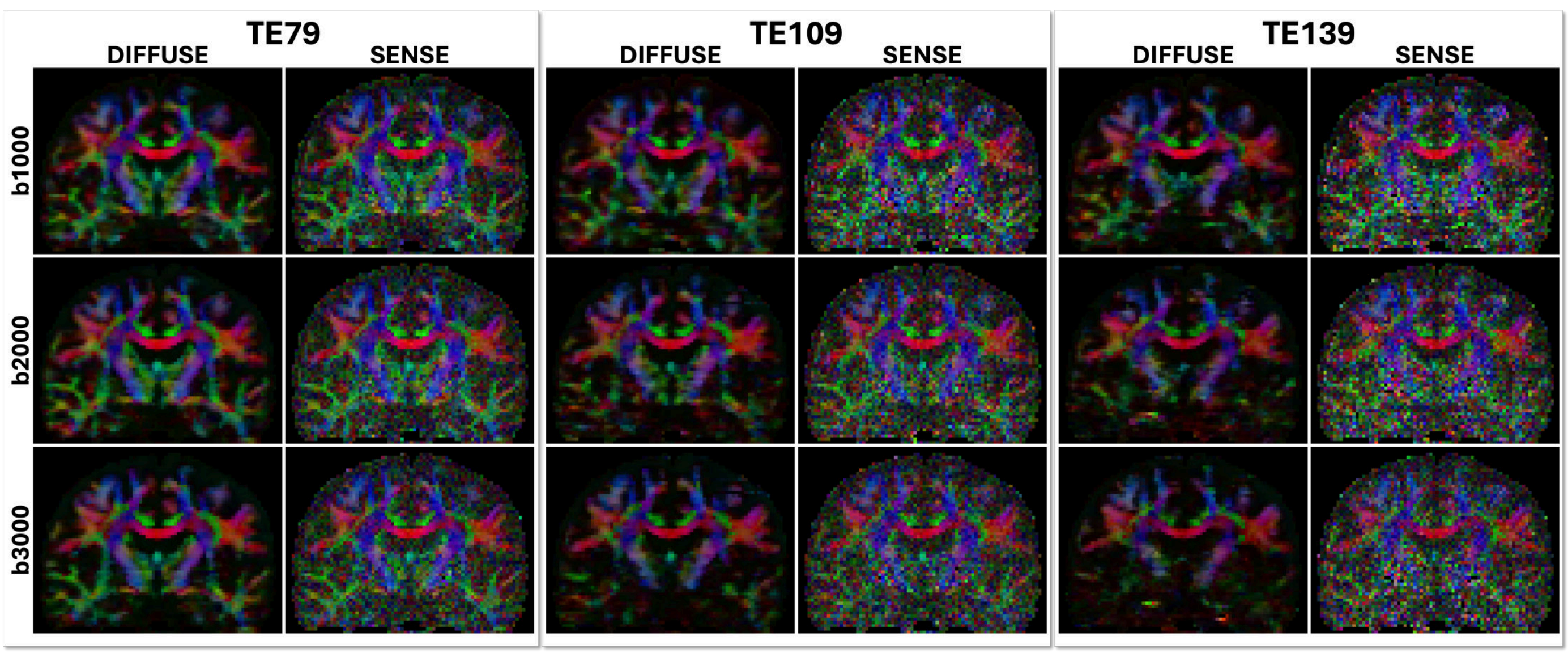

Fig. S5. DTI orientation maps at coronal slice $y = 53$ across the complete 3×3 b-value/TE grid in the multi-shell multi-TE experiment. Rows show $b = 1000$, 2000, and 3000 s/mm^2. Column pairs show TE values of 79, 109, and 139 ms, with DIFFUSE and SENSE displayed side by side within each pair.
